# Study of the Active Carbon from Used Coffee Grounds as the Active Material for a High-Temperature Stable Supercapacitor with Ionic-Liquid Electrolyte

**DOI:** 10.3390/ma13183919

**Published:** 2020-09-04

**Authors:** Marcin Biegun, Anna Dymerska, Xuecheng Chen, Ewa Mijowska

**Affiliations:** Faculty of Chemical Technology and Engineering, West Pomeranian University of Technology in Szczecin, Piastów Ave. 42, 71-065 Szczecin, Poland; anna.gabryela.dymerska@gmail.com (A.D.); xuecheng.chen@zut.edu.pl (X.C.); ewa.borowiak-palen@zut.edu.pl (E.M.)

**Keywords:** biochar, supercapacitor, ionic liquid

## Abstract

This study reveals a simple approach to recycle wasted coffee grounds into highly valuable carbon material with superior electrochemical performance. Activated carbon prepared from wasted coffee grounds has been formed via hydrothermal acidic hydrolysis followed by a KOH chemical activation at 800 ${}^{\circ }C$. To understand the electrochemical properties of the sample, a set of characterization tools has been utilized: N_2_ and CO_2_ adsorption–desorption isotherms, thermal gravimetric analysis, Fourier transform infrared spectroscopy, Raman spectroscopy and scanning electron microscopy. The specific surface area obtained from a Brunner–Emmett–Teller (BET) analysis reached 2906±19 m${}^{2}$ $g^{-1}$. Prepared sample (designated as ACG-800KOH) was tested as electrode material in an electric double layer capacitor (EDLC) device with ionic liquid PYR13-TFSI as an electrolyte. The EDLC test was conducted at temperatures ranging from 20 to 120 ${}^{\circ }C$. The specific material capacitance reached 178 F $g^{-1}$ measured at 20 ${}^{\circ }C$ and 50 A $g^{-1}$ and was in the range 182 to 285 F $g^{-1}$ at the 20 to 120 ${}^{\circ }C$ temperature range.

## 1. Introduction

Research on Electric/Electrochemical Double Layer Capacitors (EDLC), commonly called Supercapacitors, is still a key area especially in times of intensive popularization of electric vehicles around the world. They can act as a power buffer in the process of transferring energy from the main battery or fuel cell to the vehicle’s propulsion engine in the event of a sudden increase in power demand or high acceleration of the vehicle. Also, supercapacitor will be a buffer of electric energy during the regenerative braking stage of the vehicle in which the engine works as a generator, transforming the kinetic energy of the vehicle into electric current. This function can be fulfilled by EDLCs due to high power density, in the order of 10 kW ${kg}^{-1}$ or more. In contrast to batteries mostly based on Li-ION technology used as the main source of electricity in vehicles, they are characterized by high energy density but moderate power density resulting from the reaction kinetic limits [1]. Supercapacitors can be divided, in the first place, by the way the electric charge is stored. If the charge is accumulated in the electric field of the double electric layer, we refer to electric double-layer capacitors. However, in pseudocapacitors, the charge is stored by a properly selected electrode material that undergoes a fast redox reaction. According to this criterium, there are also hybrid capacitors in which one electrode is a classic electric double-layer electrode, while the other works as a Faraday redox material [2]. Classical supercapacitors have an electrode material with a very high specific surface area which are mainly carbon materials such as activated carbon [3], carbon nanotubes [4], reduced graphene oxide [5] and carbon aerogel [6]. Pseudocapacitors use metal oxides/hydroxides [7] or conductive polymers [8] as electrode active materials. Furthermore, classification of supercapacitors is based on the type of electrolyte that is used. In EDLC types with active carbon material, the first group consists of aqueous solutions of bases, acids or salts such as 1 M KOH, 1 M H_2_SO_4_, 0.5 M Na_2_SO_4_, 1 M LiCl [9] and others. The second group is organic salt solutions where the solvent, in most cases, is anhydrous acetonitrile or propylene carbonate, e.g., LiClO_4_, (C_2_H_5_)_4_ N(BF_4_) [10]. The third group is Room Temperature Ionic Liquids (RTIL) [11,12,13] or mixtures thereof. Pseudocapacitors require to use an electrolyte that is compatible with the redox material used, it can be an aqueous electrolyte, e.g., 0.5 M Na_2_SO_4_ [14] as well as an organic one, e.g., 1 M LiClO_4_ in propylene carbonate [15].

Organic electrolytes have a definite advantage of maximum energy stored in the supercapacitor over aqueous electrolytes. This is due to the equation: $E=0.5C\;\cdot \;U^{2}$, where the electric charge (C) is equal to half of the capacity (F) multiplied by the square of the maximum voltage (V) to which the capacitor can be charged [16]. However, organic electrolytes, especially those based on acetonitrile, are considered toxic when the casing of the EDLC device is unsealed and are also flammable as opposed to aqueous electrolytes. Usually, the maximum operating temperature of such a system does not exceed 65 ${}^{\circ }C$. Certain solutions can be application of Room Temperature Ionic Liquids RTIL [17]. They are non-flammable [18] and can be tested in specific applications by appropriate selection of cation and anion components [19]. Properly selected, in terms of electrochemical properties, ionic liquid should be characterized by high stability in the widest range of potentials, high ionic conductivity and a melting point below the required operating temperature of the supercapacitor. In addition, RTILs should have high-temperature stability, shifting the permitted operating range above 100 ${}^{\circ }C$. This may be crucial in some supercapacitor applications. The current state of the art on ionic liquids in EDLC, includes the impact of different types of cations and anions [11,20,21]. Theoretically, the working potential window can be even 5–6 V wide, but in practice the combination with active carbon material it is limited to 3.5–4 V [22].

The manufacturers of currently produced supercapacitors use activated carbon as the electrode material obtained by various methods that are the subjects of patents (Maxwell Technologies, Skeleton Technologies). Practically, only organic electrolytes types are used [23]. Aqueous solutions of strong acids and some salts are not chemically compatible with their technologies and materials used in the production of supercapacitors. Industrially active carbon is made from different precursors, e.g., coals, peat, wood, coconut and shells, and some synthetic polymers. This process is tailored to the given raw material [24]. The use of other precursors carries the risk of a specific spread of the final parameters of the obtained activated carbon. However, these raw materials are considered often as waste, so the cost to obtain them is very low and therefore it can offset the inconvenience associated with lower repeatability of parameters. There are numerous scientific papers reporting the use of precursors derived from natural waste sources to obtain activated carbon. One can mention, e.g., camellia oleifera shell [25], coffee beans [26], sugar cane bagasse [27], peanut shell [28], rice husk [29], pomegranate seed [30] and dead Neem leaves [31]. Various activation methods have also been used, such as chemical activation by KOH, ZnCl_2_, H_3_PO_4_, KHCO_3_, physical by CO_2_/H_2_O or the self-activation method.

**Figure 1:**
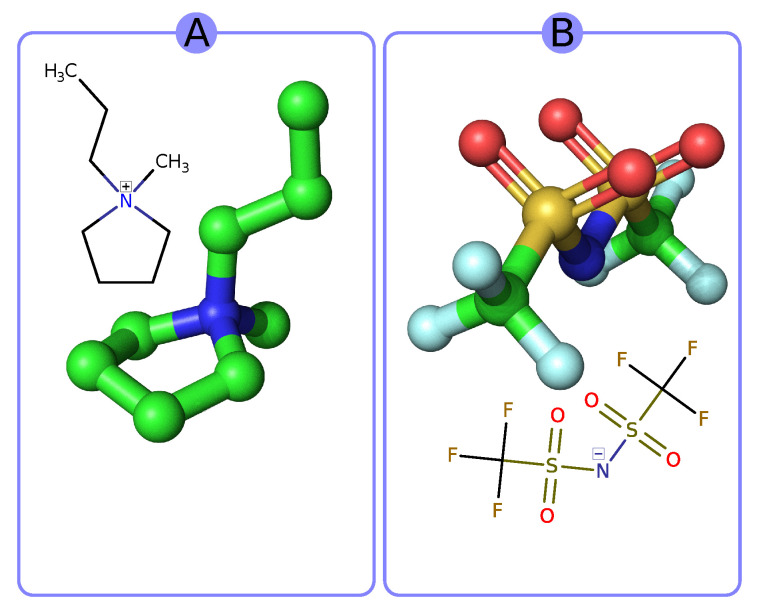
Three-dimensional visualization and structural formula of (**A**) 1-methyl-1-propylpyrrolidine and (**B**) bis(trifluoromethylsulfonyl)imide.

In this contribution, we focus on the application of activated carbon obtained through hydrothermal treatment and thermal activation with KOH of the natural waste material that is wasted coffee grounds. Additionally, we tested obtained samples as electrode material with RTIL 1-methyl-1-propyl-pyrrolidinium bis(trifluoromethylsulfonyl)imide PYR13-TFSI as the electrolyte of choice working at a broad temperature range. Its structural formula is shown in Figure 1 and the properties are presented in Table 1. It is an interesting hydrophobic type of ionic liquid due to its wide potential stability window and is currently available in decent purity from several suppliers [22,32].

## 2. Experimental

### 2.1. Materials

All common reagents were used in analytical grade without additional purification. Hydrochloric acid, sulfuric acid, potassium hydroxide and isopropanol were purchased from Avantor Performance Materials Poland S.A. (Gliwice, Poland) The carbon-coated 20 μm thick aluminum foil was purchased from MTI Corporation (Richmond, VA, USA) and TF 4350 separator from Nippon Kodoshi Corporation (Kochi, Japan). The wasted coffee grounds came from a pressure automatic espresso machine that used 100% arabica coffee from Lavazza (Turin, Italy). Wasted coffee material was collected for 3 months to obtain a good sample averaging. For all solution preparation and washing the Ultra Pure water DI was used.

### 2.2. Material Preparation

**Figure 2:**
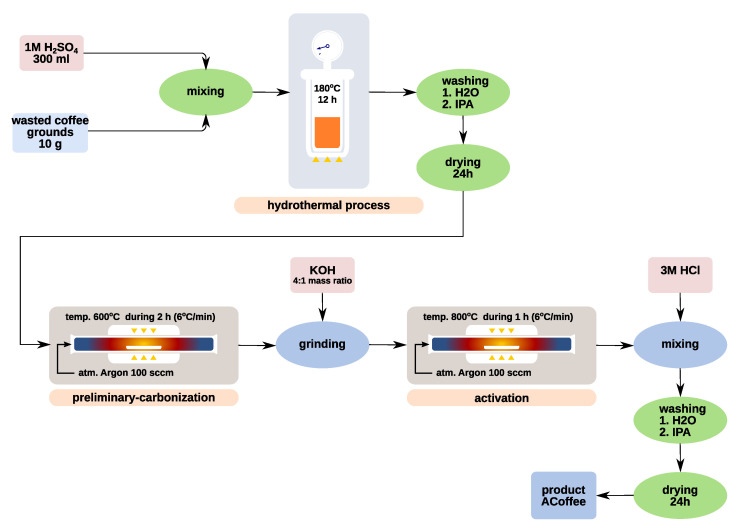
Diagram showing synthesis of active carbon from wasted coffee grounds.

Synthesis of the active carbon biochar material was conducted in a two-step process which is presented by a diagram in Figure 2. In the first stage, 10 g of the ground and dried precursor (wasted coffee grounds) was dispersed in 300 mL of 1 M sulfuric acid solution and placed in a 500 mL high-pressure reactor (autoclave) in a Teflon insert. Hydrothermal hydrolysis process was conducted for 12 h at 180 ${}^{\circ }C$. The resulting black precipitate was then washed by water and isopropanol and finally dried. In the next stage, the obtained material was preliminarily carbonized in the tube furnace EHA 12/300B (Carbolite Gero, Sheffield, UK) at 600 ${}^{\circ }C$ during 2 h with 6 ${}^{\circ }C$ $\min^{-1}$ heating rate in an inert atmosphere and then ground with KOH in a 4:1 (KOH/material) mass ratio and activated in the tube furnace at 800 ${}^{\circ }C$ during 1 h with 6 ${}^{\circ }C$ $\min^{-1}$ heating rate in an inert atmosphere. After activation, the material was dispersed in 3 M HCl. After 10 h, the material was filtered and then washed several times with water until an inert filtrate was obtained, and finally rinsed with isopropanol and dried in a vacuum oven for 24 h at 5 mbar.

### 2.3. Electrochemical Measurements

The electrode material was prepared by mechanical homogenization (35k rpm) of a mixture of activated carbon, acetylene black and binder with the addition of water in a mass ratio 75:10:15. As the binder, a water-based polyacrylic-urethane emulsion varnish (Acrylicos Vallejo S.L., Barcelona, Spain) was used. The prepared homogeneous emulsion was applied to carbon-coated aluminum foil by an airbrush gun (nozzle diameter 0.5 mm, 2 bar air pressure) with direct drying by an IR lamp (150 W, Philips Polska, Warsaw, Poland). Prepared foil with active material was cut into circle-shaped electrodes (diameter 15 mm) by a disk cutter press and dried in a vacuum oven for 24 h at 5 mbar. After weighing, all prepared electrodes were transported to a high purity glove-box and, after 24 h, assembled to a CR 2032 coin cell (MTI Corporation, Richmond, VA, USA) with doubled TF 4350 separator soaked with PYR13-TFSI electrolyte. The average active mass loading was equal to 1.63±0.19 mg per electrode and 1.85 mg cm^−2^ as surface density. Electrochemical measurements of the assembled CR 2032 cells were performed with the potentiostat/galvanostat electrochemical multichannel station VMP 3 (BioLogic Sciences Instruments, Seyssinet-Pariset, France). For the EDLC temperature test the CR 2032 cells were placed in an oven with temperature control from 20 to 200 ${}^{\circ }C$ ± 0.5 ${}^{\circ }C$.

### 2.4. Material Characterization

Electron imaging was performed by Scanning Electron Microscope VEGA-3 (TESCAN, Brno-Kohoutovice, Czech Republic). X-ray diffraction (XRD) was performed by Aeris Research edition (Malvern Panalytical Ltd., Malvern, UK) with Cu anode X-ray source. Nitrogen and Carbon Dioxide physisorption was conducted at Accelerated Surface Area and Porosimetry System ASAP 2460 (Micromeritics Instrument Corporation, Norcross, GA, USA), samples were degassed at 250 ${}^{\circ }C$ for 12 h. Thermal Gravimetric Analysis (TGA) was conducted by an SDT Q600 (TA Instruments, New Castle, DE, USA). IR spectroscopy was performed by a Fourier Transform-Infrared Spectrometer Nickolet 6700 (Thermo Fisher Scientific, Waltham, MA, USA).

## 3. Results

### 3.1. Material Characterization

The prepared sample exhibited the following yields after each stage of hydrothermal process: 25%, preliminary carbonization: 50%, activation: 50%. Overall, this gives a process yield of ≈10%.

**Figure 3:**
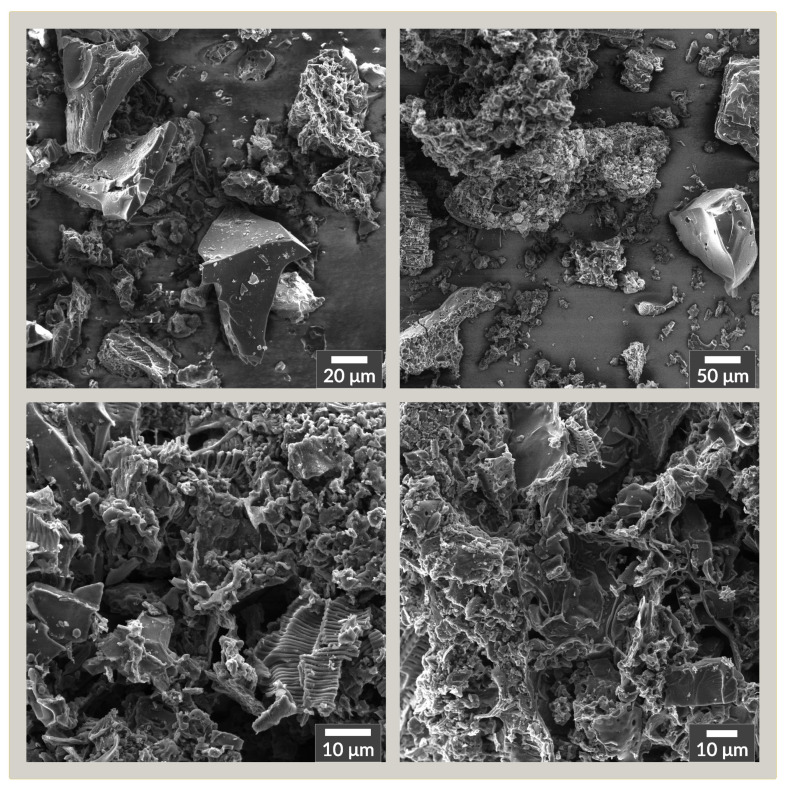
Scanning Electron Microscope images of the ACG-800KOH material with different magnifications.

Scanning Electron Microscope images of the ACG-800KOH material are presented in Figure 3. They show that the structure of the obtained carbon material consists of harder fragments with a more uniform structure and a large number of fragments with a fairly complex structure of natural plant origin. The first stage of the synthesis in which the natural precursor was treated with 1 M sulfuric acid at an elevated temperature of 180 ${}^{\circ }C$ caused acid hydrolysis of cellulose and fragments with an amorphous structure, creating a skeleton with a harder structure. It was then charred at 900 ${}^{\circ }C$ in an inert atmosphere. In the activation stage, molten KOH undergoes a series of reactions resulting in the removal of part of the carbon structure and the creation and extension of the already existing complex porous structure. Potassium hydroxide works by creating micropores and fewer mesopores as a result of the oxidation of the carbon structure, according to chemical Equations (1)–(3) [34,35].

(1) $$\begin{array}{cc} 6\mathrm{KOH}+2C & \Rightarrow 2K_{2}{\mathrm{CO}}_{3}+2K+3H_{2}↑ \end{array}$$

(2) $$\begin{array}{cc} 2\mathrm{KOH}+C+H2O & \Rightarrow K_{2}{\mathrm{CO}}_{3}+2H_{2}↑ \end{array}$$

(3) $$\begin{array}{cc} 2\mathrm{KOH}+2C & \Rightarrow 2\mathrm{CO}↑+2K+H_{2}↑ \end{array}$$

**Figure 4:**
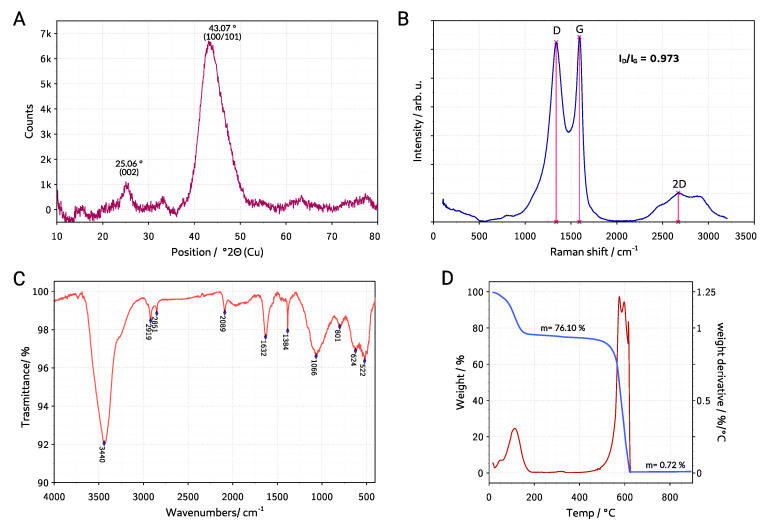
(**A**) X-ray diffraction (XRD) diffraction pattern, (**B**) Raman spectrum, (**C**) Fourier transform infrared spectroscopy (FTIR) spectrum, and (**D**) thermal gravimetric analysis (TGA) plot of the ACG-800KOH material.

The XRD diffraction pattern of the ACG-800KOH material is presented in Figure 4A. The pattern shows broad reflections of the graphite plane (002) and (100/101) at 25.06 and 43.07${}^{\circ }2\Theta$. The reflections of potassium compounds are not observed indicating efficient washing procedure. From Bragg’s law [36], it is possible to calculate the distance between planes (002) $d_{\left(002\right)}$ according to Equation (4) where $\lambda_{xrd}$ is a X-ray wavelength (for Cu $K_{\alpha }$ is equal 0.15409 nm), n is the “order” of reflection, normally assumed to be equal to 1 and $\Theta_{\left(002\right)}$ is a position of the (002) plane reflection in rad. This distance calculated for ACG-800KOH XRD diffraction pattern is equal 0.3551 nm, which is 5.9% higher than for regular graphite elementary cell (0.3354 nm).

(4) $$d_{\left(002\right)}=\frac{n\lambda_{xrd}}{2sin\left(\Theta_{\left(002\right)}\right)}$$

Additional information on the degree of graphitization of the activated carbon structure can be obtained from the Raman analysis. The Raman spectrum of the material (laser: 532 nm) is shown in Figure 4B. It shows the characteristic D (1339 cm^−1^) and G (1591cm^−1^) bands. The G band is the result of in-plane vibrations of the $sp^{2}$ bonded carbon atoms coming from a graphene plane. The D band indicates the presence of a deformed carbon structure in the material. In addition, the intensity ratio of these bands is a measure of disorder and represents the ratio of the $sp^{2}/sp^{3}$ carbon [37]. For the ACG-800KOH material that ratio is equal $I_{D}/I_{G}=0.973$. The spectrum also shows the 2D (2670 cm^−1^) band, however it has much smaller intensity than the G and D bands and it is composed of several smaller bands of different height and width, which indicate defective graphene structure.

To determine the types of functional groups present on the surface of the material, a Fourier transform infrared spectroscopy (FTIR) measurement was carried out and presented in Figure 4C. The samples were prepared as pastille using IR grade KBr in a mass ratio 300:1. The broad, strong band at 3440 cm^−1^ is related to stretching of the O-H hydroxyl functional group. The 2919 cm^−1^ and 2851 cm^−1^ bands can be attributed to the C-H stretching group as for alkane and aldehyde. The weak band at 2089 cm^−1^ corresponds to overtone of the C=C band. At 1632 cm^−1^ the stretching of C=C aromatic and C=O carbonyl groups occurs. The 1384 cm^−1^ can be attributed to oxygen-containing functional groups, for example, C=O and C-O of carboxylic groups. The relatively wide band at 1066 cm^−1^ is stretching vibration of C-O group in alcohol, phenol, ether, or ester. The 800 cm^−1^ band and below 600 cm^−1^ band with average intensity can be attributed to C=C bending vibrations. Activated carbon FTIR spectra appear to be complex due to the possibility of a large number of functional groups formed in the process of carbonization and activation [38].

In order to determine the content of inorganic residues in the material, thermogravimetric analysis was conducted in air atmosphere. The TGA curve is presented in Figure 4D. The first decrease in sample mass occurs in the temperature range from 20 to 170 ${}^{\circ }C$ and constitutes 23.9% of the total mass. This is certainly due to the desorption of accumulated moisture and gases inside the porous structure of the material. This demonstrates the excellent adsorption properties of the activated carbon and confirms that a temperature of 250 ${}^{\circ }C$ is sufficient to perform the degassing process of the sample before measuring nitrogen and carbon dioxide adsorption. The next stage of weight loss is in the range of 500 to 625 ${}^{\circ }C$ and this is assigned to oxidation and combustion of the carbon skeleton of the sample. The ash content of the sample represents less than 1 wt% of the total weight. This demonstrates high purity of the tested carbon material.

**Figure 5:**
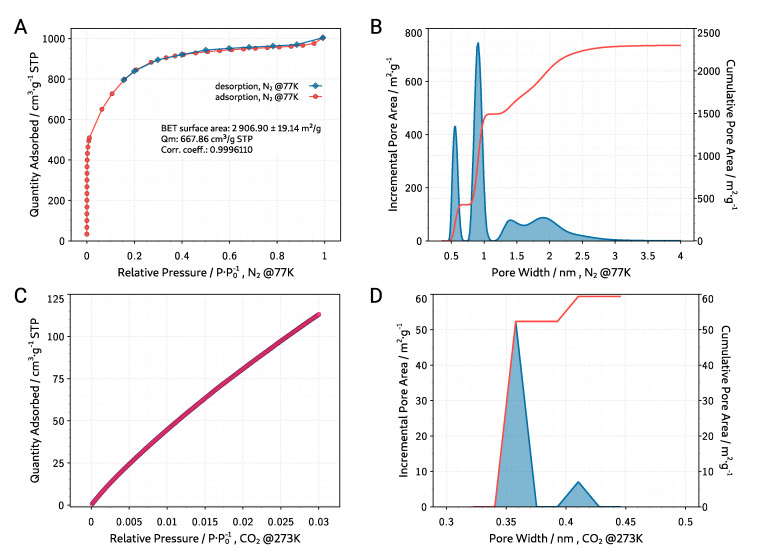
(**A**) N${}_{2}@77K$ adsorption isotherm, (**B**) cumulative and incremental pore size distribution from Non-Local Density Functional Theory (NLDFT) analysis of the N${}_{2}@77K$ adsorption, (**C**) CO${}_{2}@273K$ adsorption isotherm, (**D**) cumulative and incremental pore size distribution from NLDFT analysis of the CO${}_{2}@273K$ adsorption for the ACG-800KOH material.

Activated carbon is a porous material with a complicated and developed structure, mainly containing micropores (<2 nm) as well as mesopores (2–50 nm) and sometimes a small amount of macropores (>50 nm). One of the main techniques used to study the properties of porous activated carbons is the gas adsorption (usually nitrogen at 77 K and carbon dioxide at 273 K). Figure 5A presents the isotherm of nitrogen adsorption and desorption (at the 77 K) of the ACG-800KOH material. According to IUPAC, it represents a mixture of type I and IV isotherms [39]. However, without a clear cut-off in the high pressure $P/P_{0}$ range. A sudden increase in the curve in the low pressure $P/P_{0}$ range suggests that the material has a high content of micropores. The desorption curve shows a small hysteresis which proves the small content of mesopores in the structure of the material. The specific surface area of the carbon material was determined basing on the Brunner–Emmett–Teller theory and using MicroActive V3.01 software from Micromeritics company (Norcross, GA, USA). The determined specific surface area from the Brunner–Emmett–Teller (BET) theory is 2906±19 m${}^{2}$ $g^{-1}$ with correlation coefficient >0.9996. Non-Local Density Functional Theory (NLDFT) analysis with model “HS-2D-NLDFT, Carbon, N2, 77” using SAIEUS V3.0 software from Micromeritics company (Norcross, GA, USA) was used to determine the pore size distribution. Cumulative and incremental pore size distribution from NLDFT analysis of the $N_{2}@77K$ adsorption is presented in Figure 5B. It can be seen that the structure of the material contains micropores mostly in the ranges of 0.6 nm to 0.8 nm and less in the range of 1.3 to 2.3 nm. Calculated from NLDFT, the total pore volume is 1.44 ${\mathrm{cm}}^{3}$ $g^{-1}$ and total area of pores is 2324.09 m${}^{2}$/$g^{-1}$.

For kinetic reasons, the nitrogen adsorption method cannot be used to analyze micropores below 0.5 nm, therefore carbon dioxide sorption was also measured at 273 K. Figure 5C presents the CO${}_{2}$ adsorption isotherm (at the 273 K) made for the ACG-800KOH material. NLDFT analysis with model “CO${}_{2}$@273-Carbon, NLDFT” using MicroActive V3.01 software from the Micromeritics company (Norcross, GA, USA) was used to determine the pore size distribution. Cumulative and incremental pore size distribution from NLDFT analysis of the CO${}_{2}@273K$ adsorption is presented in Figure 5D. The analysis shows that in the micropore range below 0.5 nm the material contains mainly pores with diameter of 0.36 nm and a small number of pores with diameter of 0.41 nm.

Analysis of nitrogen and CO${}_{2}$ physiorption showed the microporous nature of the obtained activated carbon with the developed specific surface of 2900 m${}^{2}$ $g^{-1}$.

### 3.2. Electrochemical Measurements

**Figure 6:**
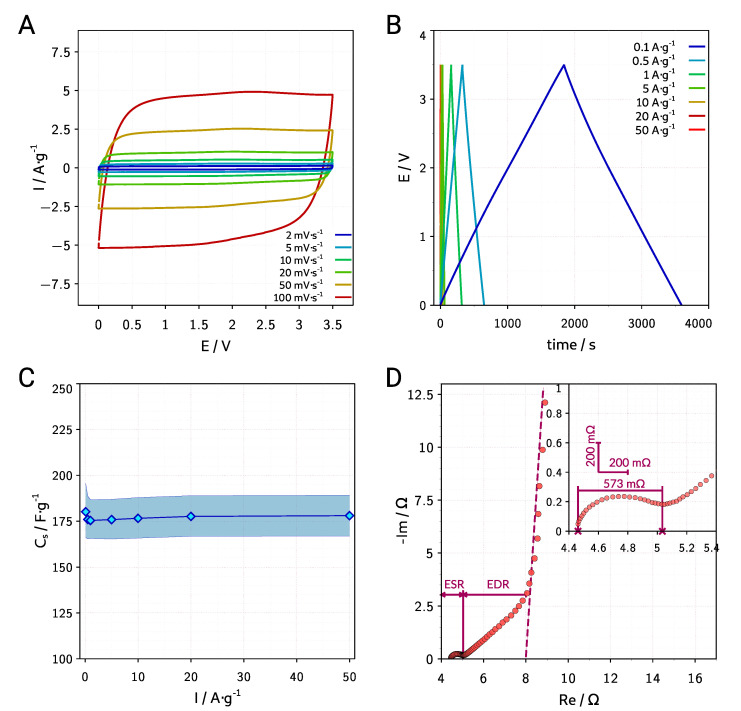
(**A**) Current–voltage (CV) plots at 2, 5, 10, 20, 50 and 100 mV $s^{-1}$ scan speed, (**B**) Galvanostatic Charging–Discharging with Potential Limitation (GCPL) at 0.1, 0.5, 1, 5, 10, 20 and 50 A $g^{-1}$ current density, (**C**) mean specific capacitance with extreme range vs. current density, (**D**) EIS at open circuit conditions for the ACG-800KOH material in a two-symmetric electrode electric double layer capacitor (EDLC) with PYR13-TFSI electrolyte.

In order to determine the performance of the obtained carbon material as an electrode material, it was tested in a two-electrode EDLC system with PYR13-TFSI as the electrolyte. The first experiment was carried out at room temperature. To examine the electrochemical characteristics the Cyclic Voltammetry CV in the 0–3.5 V voltage range and scan speed at 2, 5, 10, 20, 50 and 100 mV $s^{-1}$ was conducted and the plots are presented in Figure 6A. The charts for all scanning speeds present a shape close to a rectangle which is similar to a model supercapacitor with a purely electrostatic electric charge-accumulating characteristics [40]. The purely capacitive nature is also confirmed by the lack of clear redox peaks that could be evidence of faraday reactions between the electrode material and the electrolyte, and thus pseudo-capacitive character. Moreover, the current–voltage curves are symmetrical about the abscissa (current density), which means that the process of charging a double electrical layer at the interface between the carbon material and electrolyte, under the applied electric field, is fully reversible. This means that the capacitor has high stability of charge–discharge cycles. One of the most important EDLC tests is a Galvanostatic Charging–Discharging with Potential Limitation (GCPL). Obtained results at 0.1, 0.5, 1, 5, 10, 20 and 50 A $g^{-1}$ current densities are shown in Figure 6B. The specific capacitance $C_{s}$ ($F\;g^{-1}$) of the ACG-800KOH material was calculated at for each current density from GCPL according to Equation (5).

(5) $$C_{s}=2\frac{dQ}{0.5\;\cdot \;dU\;\cdot \;m_{el}}$$

where dQ is an electric charge (*C*) collected in capacitor within dU potential range (V), $m_{el}$ is a total electrode material mass in a device. The calculated specific capacitances for 0.1, 0.5, 1, 5, 10, 20 and 50 A $g^{-1}$ current densities are presented in Figure 6C. The $C_{s}$ was calculated as mean value with additional extremum range from 4 individual devices tested in identical conditions. The maximum value 180.1±15.0 F $g^{-1}$ of the material is obtained at the lowest current density. However, even at the highest value 50 A $g^{-1}$ it achieves 178.0±12.0 F $g^{-1}$. Such a small change in the specific capacity value along with an increase in current density indicates low resistances in the transport of ions into the pores of the carbonaceous material. This may be due to a good fit of the material’s pore size (0.8 nm) to the diameter of the electrolyte ions (0.656 nm) [33]. Electrochemical Impedance Spectroscopy measurements allows to determine the electrolyte–carbon material interface impedance properties. The Nyquist plot of the EDLC with ACG-800KOH material and PYR13TFSI as electrolyte measured at Open Circuit Potential is presented in Figure 6D. The shape of the obtained graph is suitable for material with capacitive characteristics with an initial semicircle that begins with point 4.46 Ω of the real resistance value. The semicircle endpoint 5.03 Ω specifies the Equivalent Series Resistance value. The rest of the spectrum corresponds to a lower frequency and passes into a straight line corresponding to the capacitive element. Extrapolation of the linear range to the real resistance axis allows to calculate the value of 2.97 Ω of the Equivalent Distributed Resistance EDR according to Transmission Line Theory [41].

Based on GCPL measurements of the EDLC device with ACG-800KOH material, specific energy ($E_{sp}$, $Wh\;kg^{-1}$) and specific power ($P_{sp}$, $W\;kg^{-1}$) were calculated using Equations (6) and (7).

(6) $$\begin{array}{cc} E_{sp} & =\frac{C_{sp}\;\cdot \;U^{2}}{8*m_{el}} \end{array}$$

(7) $$\begin{array}{cc} P_{sp} & =\frac{E_{sp}}{t} \end{array}$$

where $C_{sp}$ is a specific capacitance (F), *U* is working potential range (V), *t* is a discharge time (h), $m_{el}$ is a total electrode material mass in a device [42].

**Figure 7:**
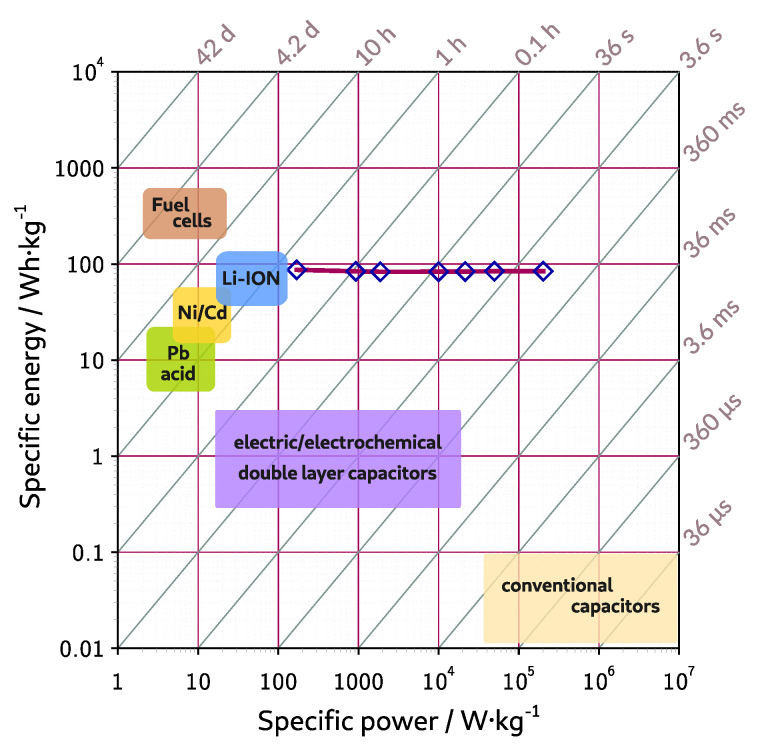
Specific energy vs. specific power calculated from GCPL measurements for the ACG-800KOH material in a two-symmetric electrode EDLC with PYR13-TFSI electrolyte.

The tested material has reached specific energy $E_{sp}$ equal to 84.10±0.83 Wh $kg^{-1}$ and specific power $P_{sp}$ in a range from 0.170 to 202 $\mathrm{kW}{\mathrm{kg}}^{-1}$. The relationship between specific energy and specific power is presented as a Ragone plot in a Figure 7.

**Figure 8:**
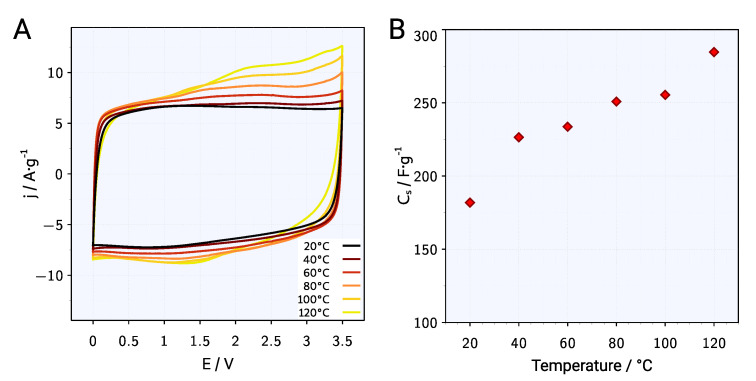
(**A**) CV plots at 20, 40, 60, 80, 100, 120 ${}^{\circ }C$ temperature with 50 mV $s^{-1}$ scan speed, (**B**) specific capacitance at 5 A $g^{-1}$ current density vs. temperature for the ACG-800KOH material in a two-symmetric electrode EDLC with PYR13-TFSI electrolyte.

In order to use the advantage of high thermal stability of the PYR13-TFSI ionic liquid, EDLC measurements were also carried out at elevated temperatures of 20, 40, 60, 80, 100 and 120${}^{\circ }C$, respectively. Cyclic Voltammetry plots of the ACG-800KOH material in a two-symmetric electrode EDLC with PYR13-TFSI electrolyte conducted with 50 mV $s^{-1}$ scan speed at each temperature are presented in Figure 8A. With the increase in the temperature, the current increases in the anode part of the CV curve, while the change in the cathode part is small. Asymmetrical increase of the CV curve in the anode range may indicate the existence of Faraday reactions between the electrolyte and the electrode material that intensify as the system operating temperature increases. In addition, this will reduce the stability of EDLC at elevated temperatures and reduce the efficiency of the charge–discharge cycle because part of the energy consumed for the charge cycle will not be returned in the discharge cycle.

The specific capacitance obtained from a GCPL measurement at 20, 40, 60, 80, 100, 120 ${}^{\circ }C$, respectively and at 5 A $g^{-1}$ current density are shown in Figure 8B. The specific capacity of the material linearly increases with the increasing operating temperature of the system, from 182 F $g^{-1}$ at 20 ${}^{\circ }C$ to 285 F $g^{-1}$ at 120 ${}^{\circ }C$. The phenomenon is caused by a decrease in the viscosity of the electrolyte-ionic liquid, which causes an increase in the electrical conductivity of the electrolyte, and thus increases the propagation of charges in the electrode material. The effect of increasing electrical conductivity for liquid electrolytes, especially ionic liquids, is confirmed experimentally [33,43,44] and on the basis of molecular modeling [45] and increases from the value 3.8 mS cm^−1^ at 25 ${}^{\circ }C$ to 10 mS cm^−1^ at 60 ${}^{\circ }C$.

## 4. Conclusions

Activated carbon was produced from coffee grounds waste as a result of a two-stage process: hydrothermal acid hydrolysis and chemical activation of KOH at 800 ${}^{\circ }C$. The resulting material was tested for use as an electrode material in an Electrochemical Double Layer Capacitor at normal and elevated temperature. The resulting porous material is an activated carbon with a high surface development, Specific Surface Area was equal to 2906±19 m${}^{2}$ $g^{-1}$, and has micropores in the range of 0.6–0.8 nm. It shows high electrochemical efficiency in combination with a PYR13-TFSI ionic liquid as an electrolyte and Specific Capacitance equal to 178.0±12.0 F $g^{-1}$ at 50 A $g^{-1}$. The obtained material reached maximum specific energy $E_{sp}$ and specific power $P_{sp}$ parameters equal to, respectively, 84 Wh $kg^{-1}$ and 202 kW $kg^{-1}$. In addition, the operating temperature range of such a system significantly exceeds the range of traditionally used organic electrolytes based on acetonitrile. Specific Capacitance calculated from a GCPL test at 120 ${}^{\circ }C$ was equal to 285 F $g^{-1}$ at 5 A $g^{-1}$. This is very desirable for the safety of the use of supercapacitors and, in addition, extends the operating temperature range for these devices.

## Figures and Tables

**Table 1:** PYR13-TFSI ionic liquid properties.

Symbol	Melting Point $/{}^{\circ }C$	Viscosity /mPas	Conductivity $/S\;\cdot \;{\mathrm{cm}}^{-1}$	Ref.
PYR13-TFSI	12	63	$3.8\times 10^{-3}$	[33]

## References

[1] Simon P., Gogotsi Y. (2009). Materials for Electrochemical Capacitors. Nanoscience and Technology.

[2] Sudhakar Y.N., Selvakumar M., Bhat D.K. (2018). Biopolymer Electrolytes: Fundamentals and Applications in Energy Storage.

[3] Pandolfo A.G., Hollenkamp A.F. (2006). Carbon Properties and Their Role in Supercapacitors. J. Power Sources.

[4] Niu C., Sichel E.K., Hoch R., Moy D., Tennent H. (1997). High Power Electrochemical Capacitors Based on Carbon Nanotube Electrodes. Appl. Phys. Lett..

[5] El-Kady M.F., Kaner R.B. (2013). Scalable Fabrication of High-Power Graphene Micro-Supercapacitors for Flexible and on-Chip Energy Storage. Nat. Commun..

[6] Kim S.J., Hwang S.W., Hyun S.H. (2005). Preparation of Carbon Aerogel Electrodes for Supercapacitor and Their Electrochemical Characteristics. J. Mater. Sci..

[7] Hoang T.T.B., Mohapatra S., Nguyen T.A., Nguyen-Tri P. (2019). Chapter 26-Noble Metal–Manganese Oxide Nanohybrids Based Supercapacitors. Noble Metal-Metal Oxide Hybrid Nanoparticles.

[8] Snook G.A., Kao P., Best A.S. (2011). Conducting-Polymer-Based Supercapacitor Devices and Electrodes. J. Power Sources.

[9] Ramachandran R., Wang F. (2017). Electrochemical Capacitor Performance: Influence of Aqueous Electrolytes. Supercapacit. Theor. Pract. Solut..

[10] Yang C., Sun M., Wang G., Cheng Q., Bao H., Li X., Saha N., Saha P. (2017). High Energy-Density Organic Supercapacitors Based on Optimum Matching between GNS/aMWCNT@polyaniline Nanocone Arrays Cathode and GNS/aMWCNT@poly(1,5-Diaminoanthraquinone) Nanoparticles Anode. Chem. Eng. J..

[11] Mousavi M.P.S., Wilson B.E., Kashefolgheta S., Anderson E.L., He S., Bühlmann P., Stein A. (2016). Ionic Liquids as Electrolytes for Electrochemical Double-Layer Capacitors: Structures That Optimize Specific Energy. ACS Appl. Mater. Interfaces.

[12] Lewandowski A., Galiński M. (2004). Carbon–Ionic Liquid Double-Layer Capacitors. J. Phys. Chem. Solids.

[13] Sakaebe H., Matsumoto H. (2003). N-Methyl-N-Propylpiperidinium Bis(Trifluoromethanesulfonyl)Imide (PP13–TFSI) – Novel Electrolyte Base for Li Battery. Electrochem. Commun..

[14] Yu L., Gan M., Ma L., Huang H., Hu H., Li Y., Tu Y., Ge C., Yang F., Yan J. (2014). Facile Synthesis of MnO2/Polyaniline Nanorod Arrays Based on Graphene and Its Electrochemical Performance. Synth. Met..

[15] Brezesinski T., Wang J., Tolbert S.H., Dunn B. (2010). Ordered Mesoporous *α*-MoO3 with Iso-Oriented Nanocrystalline Walls for Thin-Film Pseudocapacitors. Nat. Mater..

[16] Burke A. (2007). R&D Considerations for the Performance and Application of Electrochemical Capacitors. Electrochim. Acta.

[17] Fernicola A., Scrosati B., Ohno H. (2006). Potentialities of Ionic Liquids as New Electrolyte Media in Advanced Electrochemical Devices. Ionics.

[18] Kurc B., Siwińska-Stefańska K., Jakóbczyk P., Jesionowski T. (2016). Titanium Dioxide/Graphene Oxide Composite and Its Application as an Anode Material in Non-Flammable Electrolyte Based on Ionic Liquid and Sulfolane. J. Solid State Electrochem..

[19] Stepnowski P., Letcher T.M. (2007). Chapter 16—Sorption, Lipophilicity and Partitioning Phenomena of Ionic Liquids in Environmental Systems. Thermodynamics, Solubility and Environmental Issues.

[20] Lewandowski A., Olejniczak A., Galinski M., Stepniak I. (2010). Performance of Carbon–Carbon Supercapacitors Based on Organic, Aqueous and Ionic Liquid Electrolytes. J. Power Sources.

[21] Zhang S., Brahim S., Maat S. (2018). High-Voltage Operation of Binder-Free CNT Supercapacitors Using Ionic Liquid Electrolytes. J. Mater. Res..

[22] Howlett P.C., Izgorodina E.I., Forsyth M., MacFarlane D.R. (2006). Electrochemistry at Negative Potentials in Bis(Trifluoromethanesulfonyl)Amide Ionic Liquids. Z. Phys. Chem..

[23] Balducci A. (2016). Electrolytes for High Voltage Electrochemical Double Layer Capacitors: A Perspective Article. J. Power Sources.

[24] Marsh H., Rodríguez-Reinoso F., Marsh H., Rodríguez-Reinoso F. (2006). CHAPTER 1-Introduction to the Scope of the Text. Activated Carbon.

[25] Zhang J., Gong L., Sun K., Jiang J., Zhang X. (2012). Preparation of Activated Carbon from Waste Camellia Oleifera Shell for Supercapacitor Application. J. Solid State Electrochem..

[26] Rufford T.E., Hulicova-Jurcakova D., Zhu Z., Lu G.Q. (2008). Nanoporous Carbon Electrode from Waste Coffee Beans for High Performance Supercapacitors. Electrochem. Commun..

[27] Rufford T.E., Hulicova-Jurcakova D., Khosla K., Zhu Z., Lu G.Q. (2010). Microstructure and Electrochemical Double-Layer Capacitance of Carbon Electrodes Prepared by Zinc Chloride Activation of Sugar Cane Bagasse. J. Power Sources.

[28] Wu M.b., Li R.c., He X.j., Zhang H.b., Sui W.b., Tan M.h. (2015). Microwave-Assisted Preparation of Peanut Shell-Based Activated Carbons and Their Use in Electrochemical Capacitors. New Carbon Mater..

[29] Le Van K., Luong Thi T.T. (2014). Activated Carbon Derived from Rice Husk by NaOH Activation and Its Application in Supercapacitor. Prog. Nat. Sci. Mater. Int..

[30] Okman I., Karagöz S., Tay T., Erdem M. (2014). Activated Carbons from Grape Seeds by Chemical Activation with Potassium Carbonate and Potassium Hydroxide. Appl. Surf. Sci..

[31] Biswal M., Banerjee A., Deo M., Ogale S. (2013). From Dead Leaves to High Energy Density Supercapacitors. Energy Environ. Sci..

[32] Zaripov Z.I., Gumerov F.M., Khairutdinov V.F., Małgorzata M., Zorębski E., Dzida M., Abdulagatov I.M. (2019). Thermal Conductivity and Thermal Diffusivity of Pyrrolidinium-BasedIonic Liquids at Atmospheric Pressure. Fluid Phase Equilib..

[33] MacFarlane D.R., Meakin P., Sun J., Amini N., Forsyth M. (1999). Pyrrolidinium Imides: A New Family of Molten Salts and Conductive Plastic Crystal Phases. J. Phys. Chem. B.

[34] Serafin J., Baca M., Biegun M., Mijowska E., Kaleńczuk R.J., Sreńscek-Nazzal J., Michalkiewicz B. (2019). Direct Conversion of Biomass to Nanoporous Activated Biocarbons for High CO_2_ Adsorption and Supercapacitor Applications. Appl. Surf. Sci..

[35] Tseng R.L., Tseng S.K., Wu F.C., Hu C.C., Wang C.C. (2008). Effects of Micropore Development on the Physicochemical Properties of KOH-Activated Carbons. J. Chin. Inst. Chem. Eng..

[36] Bragg W.L., Bragg W.H. (1913). The Structure of Some Crystals as Indicated by Their Diffraction of X-Rays. Proc. R. Soc. Lond. Ser. Contain. Pap. Math. Phys. Character.

[37] Ali G.A., Makhlouf S.A., Yusoff M.M., Chong K.F. (2015). Structural and Electrochemical Characteristics of Graphene Nanosheets as Supercapacitor Electrodes. Rev. Adv. Mater. Sci..

[38] Saka C. (2012). BET, TG–DTG, FT-IR, SEM, Iodine Number Analysis and Preparation of Activated Carbon from Acorn Shell by Chemical Activation with ZnCl2. J. Anal. Appl. Pyrolysis.

[39] Thommes M., Kaneko K., Neimark A.V., Olivier J.P., Rodriguez-Reinoso F., Rouquerol J., Sing K.S.W. (2015). Physisorption of Gases, with Special Reference to the Evaluation of Surface Area and Pore Size Distribution (IUPAC Technical Report). Pure Appl. Chem..

[40] Conway B.E., Pell W.G. (2003). Double-Layer and Pseudocapacitance Types of Electrochemical Capacitors and Their Applications to the Development of Hybrid Devices. J. Solid State Electrochem..

[41] Conway B.E. (2013). Electrochemical Supercapacitors: Scientific Fundamentals and Technological Applications.

[42] Fic K., Platek A., Piwek J., Frackowiak E. (2018). Sustainable Materials for Electrochemical Capacitors. Mater. Today.

[43] Liu K., Zhou Y.X., Han H.B., Zhou S.S., Feng W.F., Nie J., Li H., Huang X.J., Armand M., Zhou Z.B. (2010). Ionic Liquids Based on (Fluorosulfonyl)(Pentafluoroethanesulfonyl)Imide with Various Oniums. Electrochim. Acta.

[44] Nicotera I., Oliviero C., Henderson W.A., Appetecchi G.B., Passerini S. (2005). NMR Investigation of Ionic Liquid-LiX Mixtures: Pyrrolidinium Cations and TFSI^-^ Anions. J. Phys. Chem. B.

[45] Borodin O., Smith G.D. (2006). Structure and Dynamics of *N*-Methyl-*N*-Propylpyrrolidinium Bis(Trifluoromethanesulfonyl)Imide Ionic Liquid from Molecular Dynamics Simulations. J. Phys. Chem. B.

